# Perception and appropriation of a web-based recovery narratives intervention: qualitative interview study

**DOI:** 10.3389/fdgth.2024.1297935

**Published:** 2024-02-14

**Authors:** Yasmin Ali, Stefan Rennick-Egglestone, Joy Llewellyn-Beardsley, Fiona Ng, Caroline Yeo, Donna Franklin, Elvira Perez Vallejos, Dror Ben-Zeev, Yasuhiro Kotera, Mike Slade

**Affiliations:** ^1^ School of Health Sciences, Institute of Mental Health, University of Nottingham, Nottingham, United Kingdom; ^2^ Department of Architecture and Built Environment, Faculty of Engineering, University of Nottingham, Nottingham, United Kingdom; ^3^ NEON Lived Experience Advisory Panel, Nottingham, United Kingdom; ^4^ School of Health and Related Research, University of Sheffield, Sheffield, United Kingdom; ^5^ School of Medicine, University of Nottingham, Nottingham, United Kingdom; ^6^ School of Computer Science, University of Nottingham, Nottingham, United Kingdom; ^7^ Department of Psychiatry and Behavioral Sciences, School of Medicine, University of Washington, Seattle, WA, United States; ^8^ Center for Infectious Disease Education and Research, Osaka University, Osaka, Japan; ^9^ Faculty of Nursing and Health Sciences, Health, and Community Participation Division, Nord University, Namsos, Norway

**Keywords:** digital health intervention, online intervention, psychosis, recovery narrative, recovery story, lived experience narrative, autobiography, NEON Intervention

## Abstract

**Introduction:**

Mental health recovery narratives are widely available to the public, and can benefit people affected by mental health problems. The NEON Intervention is a novel web-based digital health intervention providing access to the NEON Collection of recovery narratives. The NEON Intervention was found to be effective and cost-effective in the NEON-O Trial for people with nonpsychosis mental health problems (ISRCTN63197153), and has also been evaluated in the NEON Trial for people with psychosis experience (ISRCTN11152837). We aimed to document NEON Intervention experiences, through an integrated process evaluation.

**Methods:**

Analysis of interviews with a purposive sample of intervention arm participants who had completed trial participation.

**Results:**

We interviewed 34 NEON Trial and 20 NEON-O Trial participants (mean age 40.4 years). Some users accessed narratives through the NEON Intervention almost daily, whilst others used it infrequently or not at all. Motivations for trial participation included: exploring the NEON Intervention as an alternative or addition to existing mental health provision; searching for answers about mental health experiences; developing their practice as a mental health professional (for a subset who were mental health professionals); claiming payment vouchers. High users (10 + narrative accesses) described three forms of appropriation: distracting from difficult mental health experiences; providing an emotional boost; sustaining a sense of having a social support network. Most participants valued the scale of the NEON Collection (*n* = 659 narratives), but some found it overwhelming. Many felt they could describe the characteristics of a *desired narrative* that would benefit their mental health. Finding a narrative meeting their desires enhanced engagement, but not finding one reduced engagement. Narratives in the NEON Collection were perceived as authentic if they acknowledged the difficult reality of mental health experiences, appeared to describe real world experiences, and described mental health experiences similar to those of the participant.

**Discussion:**

We present recommendations for digital health interventions incorporating collections of digital narratives: (1) make the scale and diversity of the collection visible; (2) provide delivery mechanisms that afford appropriation; (3) enable contributors to produce authentic narratives; (4) enable learning by healthcare professionals; (5) consider use to address loneliness.

## 1 Introduction

Narratives describing recovery from mental health problems are readily available to the public. Whilst many are presented as written autobiographies (1), we have also encountered forms such as visual art, poetry, audio recordings, video recordings, and a mixture of modalities in one narrative (2). Llewellen-Beardsley (3) has argued that mental health recovery narratives are a distinctive genre, and Llewellyn-Beardsley and colleagues (4) have advocated for an inclusive recovery narrative definition, which encompasses narratives expressing the struggles and/or adversities that a narrator has experienced, alongside their strengths, successes, and survival. This is congruent with a longstanding orientation in which the term “recovery” is defined as “a way of living a satisfying, hopeful, and contributing life even within the limitations caused by illness” (5). Whilst our focus is mental health recovery narratives, we are aware that recovery narratives are regularly published to describe a range of health experiences, including for diabetes (6) and cancer recovery (7).

Recovery narratives have been used to create individual and societal benefits. A systematic review on the uses of mental health recovery narratives (8) identified 27 different uses, which were categorised as political (e.g., supporting policy change), societal (e.g., reducing mental health stigma), community (e.g., drawing attention to mental health concerns in a particular community), service level (e.g., improving mental health and social care services), and individual (e.g., as a therapeutic tool in an individual intervention). In mental health, some Digital Health Interventions (DHIs) have included recovery narratives as a proposed *active ingredient*, e.g., one of the proposed mechanisms by which the DHI improves outcome. For example, the Self-Management And Recovery Technology (SMART) study developed a web-based intervention presenting videos in which people with lived experiences of psychosis reflected on their recovery, alongside other supportive material such as health information (9). An interview study found that SMART access helped recipients who had experienced psychosis to gain a renewed belief in achieving recovery. Accessing SMART also helped participants to feel less alone, and more connected, hopeful, and inspired (9). Recovery narratives have also been used in interventions outside of mental health (10–12), including in a randomised controlled trial (RCT) which found that self-efficacy in achieving weight loss significantly increased in participants who had read indexed narratives compared to a control group (11).

As well as being available individually, recovery narratives can be grouped together into printed or online collections, often grouped by topic. For example, a published book of psychosis recovery narratives sought to create hope for the future, for people experiencing psychosis, and for those who offer care to them (13). National anti-stigma campaigns such as Time to Change in the UK (14) and Here to Help in Canada (15) have provided web-based access to large collections of recovery narratives with the intention of reducing mental health stigma (15). In the UK, it has become a common practice for units in the National Health Service (NHS) to share collections of service user recovery narratives, for example to support the understanding of mental health conditions and recovery by other service users (16). The people who produce narrative collections can be thought of as active participants in the selection, processing, and organisation of the collection (17), and hence can influence how mental health is seen and understood (2). In interview study with collection organisers has shown that narratives are often selected to create a desired influence (8).

### 1.1 The narrative experiences online intervention

The Narrative Experiences Online (NEON) study https://www.researchintorecovery.com/research/neon has developed the NEON Intervention, a web-based DHI providing access to a collection of 659 international mental health recovery narratives, donated by individuals and from existing collections (18). Each narrative in the NEON Collection is characterized using the 77-item Inventory of Characteristics of Recovery Stories (INCRESE) (19). The development process for the NEON Intervention began by establishing a verifiable theory (20) on the characteristics (4, 21) and recipient impact (22–25) of recovery narratives. A prototype web-application was co-produced, and then iteratively improved through a feasibility study with mental health service users, including through interviews collecting immediate responses to prototype features, and reflective responses after one month of use (20). Safety strategies were developed with academic and lived experience advice (20). They included the use of content warnings (26) before access to narratives containing content with the potential to cause distress, the ability to block (and unblock) individual narratives, an information page presenting both service signposting and self-management information, and the ability to rapidly exit the NEON Intervention (for example, if participants feared stigma when using the NEON Intervention in a public place). As we developed the NEON Intervention, we were conscious that first contact with a healthcare technology can be challenging for people experiencing mental health problems (27). Hence to support new users to learn about the NEON Intervention, each participant was shown a narrative from a small set of narratives empirically identified as hope-inspiring for participants in a previous study (20), immediately after all online trial procedures and a personal profile had been completed. Participant format preferences recorded in the personal profile were respected e.g., a video first narrative was shown to participants not wanting text-based narratives. This was to aid inclusion for people experiencing difficulties processing specific narrative forms, e.g., dyslexia.

**Figure 1:**
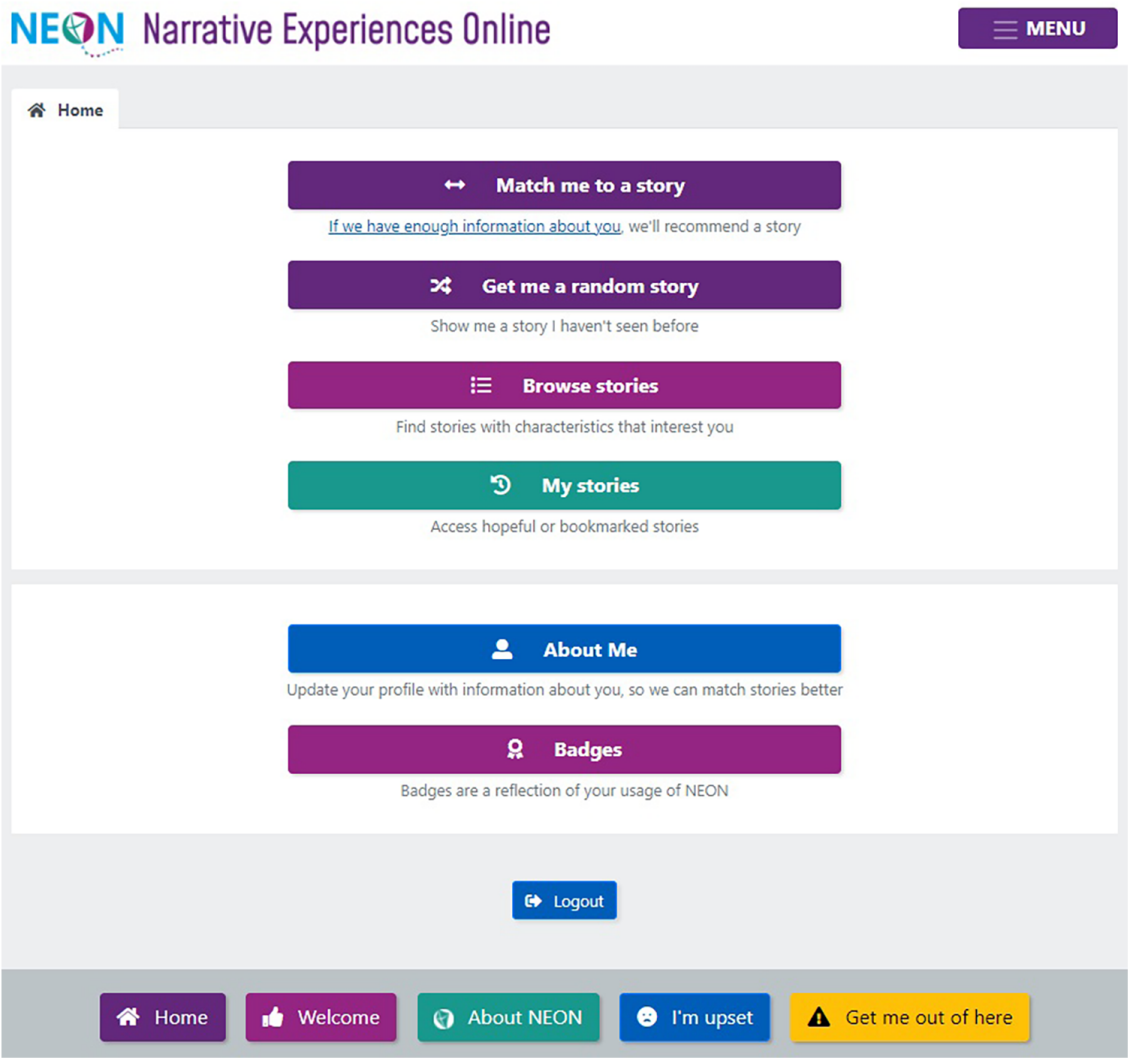
NEON Intervention home page.

**Figure 2:**
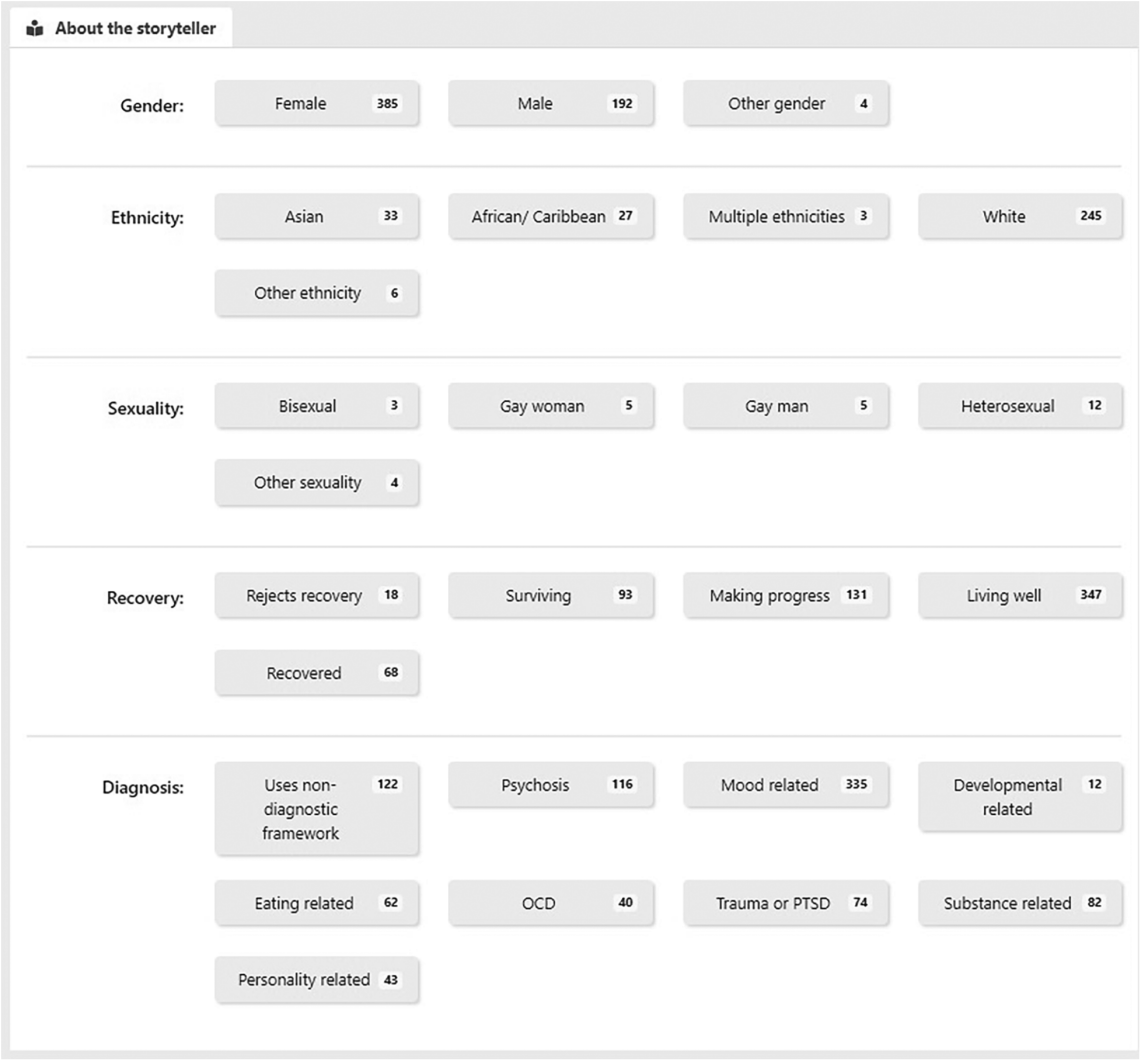
Characteristics of the narrator that can be used to filter for narratives.

**Figure 3:**
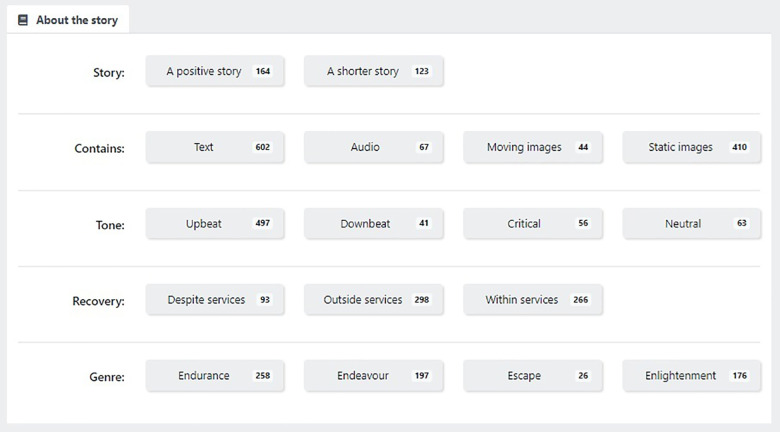
Characteristics of the narrative that can be used to filter for narratives.

The central feature of the NEON Intervention is a homepage providing users with five narrative access mechanisms (Figure 1):

(1) Match me to a story: requests that a recommender system selects a narrative. Recommender systems are algorithms designed to match digital media material to users (28).
(2) Get me a random story: requests a randomly selected narrative.
(3) Browse stories: enables a user to browse NEON Collection narratives, by selecting from categories relating to characteristics of the narrator (Figure 2) or narrative content (Figure 3).
(4) My stories—bookmarked: enables a user to request a previously-saved narrative
(5) My Stories—hopeful: enables a user to request a previously viewed narrative which they rated as high on hopefulness.

NEON has conducted two pragmatic randomised controlled trials of the NEON Intervention with different populations, which have been designed to produce a definitive result (18, 29). The NEON-O Trial included participants who experienced mental health problems other than psychosis in the last 5 years (*N* = 1,023, https://www.isrctn.com/ISRCTN63197153). It found that NEON Intervention access was effective at increasing quality of life, increasing the presence of meaning in life, and was cost-effective from the perspective of the statutory health and social care system in England (30). The NEON Trial (*N* = 739, https://www.isrctn.com/ISRCTN11152837) included participants experiencing current mental health distress, and who had experienced psychosis in the last 5 years. An evaluation of effectiveness and cost-effectiveness will be reported elsewhere. DHIs can extend health service provision to people not currently using services, and hence for external validity, both trials recruited people who had used mental health services and those who had not (31).

Prior research in mental health has established the importance of documenting user experiences of DHIs (32), for example to generate knowledge to inform iterative development of the same and related DHIs. The aim of the current study was therefore to understand the experiences of NEON Trial and NEON-O Trial participants in registering for the NEON Trial and NEON-O Trial, and in using the NEON Intervention. The objectives for the analysis presented in this paper are:

1. to identify motivations for trial participation and intervention usage
2. to document how the NEON Intervention was appropriated
3. to document perceptions of the NEON Collection that influenced usage

Our selection of these foci was informed by prior user experience and DHI evaluation research. How to create technologies that motivate engagement has been identified as a critical topic for DHI developers with the potential to influence how DHIs are used (33). Appropriation refers to the processes by which newly acquired technologies become integrated into the everyday lives of their users (34), sometimes becoming a normal feature of those lives (35, 36), and hence effectively becoming “invisible” (37). A better understanding of how interventions are appropriated can identify significant impacts and uncover unanticipated mechanisms underpinning success (38), providing developers with knowledge to guide iterative development work (39). Perceptions of DHIs can shape user experiences of those DHIs, and ultimately might influence their effectiveness (27).

## 2 Materials and methods

This paper reports an analysis of interviews collected during the process evaluation for the NEON Trial and NEON-O Trial. Ethical approval was granted in advance by Leicester Central Research Ethics Committee (REC reference: 19/EM/0326). Trial participants provided online consent when registering for the trials. All participants verbally re-confirmed their consent to take part in the interview. A description of resources allocated to trial recruitment work has been reported (31).

### 2.1 Sample

Shared trial inclusion criteria assessed at baseline were: resident in England; aged 18 or over at the time of registration; capable of accessing the Internet either on a personal computer, mobile, or device; able to understand written and spoken English; capable of providing informed consent; experience of mental health distress in the last 6 months, as assessed through three items from the Threshold Assessment Grid (40). NEON Trial participants had experience of psychosis in the last 5 years, and NEON-O Trial participants had experience of mental health problems other than psychosis in the last 5 years (both self-identified). Participants eligible for process evaluation interviews had been randomised to the intervention group; had provided all needed primary endpoint measures *or* were 32 or more days late in providing those measures (the lateness window was 31 days); and had provided consent to contact for interview through the informed consent form. All trial participants were recruited between 9th March 2020, and 26th March 2021. This coincided with a period in which the UK government imposed substantial restrictions on social interaction and physical mobility due to the COVID-19 pandemic, which were sustained throughout much of the trial follow-up period. Participants providing data late were included in case there was an association between low usage of the NEON Intervention and late provision of outcome measures, e.g., so as not to inadvertently exclude low users, who were seen as an important group to interview about their trial experiences.

An initial invitation to participate in the process evaluation was sent by email to an inception cohort of eligible trial participants, and respondents were interviewed. Work to invite participants for interview subsequently targeted a purposive sample with variation on three dimensions selected for their relevance to the user experience. These were (1) trial allocation; (2) intervention usage level; (3) mental health service usage (henceforth “service usage”). The latter was included because significant differences in baseline clinical and demographic characteristics were found between service users and non-service users in both trials, and because service use history might influence how digital interventions are perceived and used (31).

For **Trial allocation**, participants recruited were either a part of the NEON Trial or the NEON-O Trial.

For **Intervention usage level**, a maximum variation sample was sought (41). Participants were categorised into disjoint groups, using logs of unique narratives requested between first access and the primary endpoint (52 weeks). Groups covered the entire range of usage:

Group 2.A: 0–1 narrative requests
Group 2.B: 2–9 narrative requests
Group 2.C: 10–30 narrative requests
Group 2.D: 31–79 narrative requests
Group 2.E: 80+ narrative requests

Since users are shown one narrative on gaining access intervention access, then membership of group 2.A indicates no intervention usage beyond the initial registration process. Low users were defined as members of 2.A or 2.B., and high users as members of 2.C, 2.D, or 2.E.

For **service usage**, a maximum variation sample was sought. Participants were categorised according to their use of statutory mental health services, as provided in England. Historical service use was collected at baseline on a web-based demographic form, and in-trial service use was collected at the primary endpoint through an abridged Client Service Receipt Inventory (CSRI). Forms are in Supplementary Appendix 1. Groups were:

Group 3.A (no mental health service treatment). Included participants who reported no mental health service usage of any kind before and during the trial.

Group 3.B (primary care mental health service treatment only). Included participants who had received treatment from primary care mental health services before the trial, and who did not initiate specialist mental health services before or during trial.

Group 3.C (specialist mental health treatment initiated during trial). Included participants who had no specialist mental health treatment before the trial but who initiated it during the trial.

Group 3.D (specialist mental health treatment before trial). Included participants who had received lifetime treatment from mental health specialist services before the trial.

### 2.2 Procedure

Semi-structured interviews were conducted with participants using Microsoft Teams or by telephone. Pilot interviews were conducted with members of the NEON study Lived Experience Advisory Panel (*n* = 5) to assess interview procedures, and refinements were made, e.g., giving participants the option to turn off their video if preferred. All interviews were audio-recorded using an encrypted recording device. Additionally, Microsoft Teams interviews were recorded using its recording function. Participants were offered £20 as compensation for their time and effort.

The topic guide requested participants to reflect on their year-long experience of using the NEON Intervention. A spreadsheet with summary information about their NEON Intervention usage up to the day before the interview was shown to each participant to aid reflection. During the interview, participants were asked to reflect on how relevant and inclusive the NEON Collection was. Participants were asked to describe how they had appropriated the NEON Intervention and to describe any impacts they felt had resulted from its use. Researcher prompts were used to support further questioning, and to aid participants explaining their responses in places. Regular meetings were held by research team members to revise the topic guide. A sample of the topic guide is presented in Supplementary Appendix 2.

Most interview recordings were transcribed by the research team, working from an initial Microsoft Teams auto-transcription where available. Some interviews with difficult intelligibility were transcribed professionally. All transcripts were pseudonymised and checked for accuracy by researchers before analysis. Participant names were replaced with fictional names, and named locations were redacted.

### 2.3 Analysis

An inductive thematic analysis was conducted using QSR International NVivo Version 12 Pro. The analyst team consisted of six members with various disciplinary backgrounds, some with personal experience of mental health problems. A preliminary coding framework was established through parallel coding of three transcripts. YA integrated codes produced by individual analysts into a coherent framework. The coding framework was then refined through the analysis of 32 further transcripts by YA, with qualitative analyst meetings held at critical points in this process as selected by YA, to discuss the analysis of transcripts, compare findings, and enrich developing codes and themes. Due to approaching saturation, the remaining 19 transcripts were examined for discrepant content only (e.g., content not already accounted for in existing codes), and the coding framework refined where this was identified. Nodes with the greatest relevance for understanding trial behaviours and supporting future intervention development work were selected and described. Nodes describing the impact of the NEON Intervention will be reported elsewhere. Selected descriptive characteristics of participants who were interviewed were tabulated. Ethnicity responses were grouped into two disjoint categories (White British, racialised ethnicity) to avoid risk of self-identification due to small numbers of participants in most categories other than White British, following UK Data Service guidance (42). Gender categories were Female; Male; Other.

## 3 Results

Fifty-four trial participants were interviewed. Most interviews were conducted using Microsoft Teams (*n* = 49), but five participants chose a telephone interview (*n* = 5). Interviews lasted between 45 and 116 min. Participant characteristics are presented in Table 1.

**Table 1:** Participant characteristics and group allocation.

Characteristic	*N*
Total Participants	*N* = 54
Gender	Female: 30
	Male: 21
	Other: 3
Age	
Mean (SD)	40.4 (12.2)
Median (min, max)	40 (21,72)
Ethnicity	White British: 46
	Minoritized ethnicity: 8
Trial Allocation (Dimension 1)	NEON Trial: 34
	NEON-O Trial: 20
Intervention Usage level (Dimension 2)	Low user: 23
	2.A (0–1 narratives): 5
	2.B (2–9 narratives): 18
	High User: 31
	2.C (10–30 narratives): 12
	2.D (31–79 narratives): 13
	2.E (80 + narratives): 6
Health service usage (Dimension 3)	3.A (No mental health service treatment): 0
	3.B (Primary care mental health service treatment only): 2
	3.C (Specialist mental health treatment initiated during trial): 0
	3.D (Specialist mental health treatment before trial): 52

Ninety nine trial participants met the criteria for category 3.A (31), and yet none were successfully recruited for interview, despite a disproportionately large effort to recruit people into this important purposive sampling category. We conclude that participants who had received no mental health service treatment were harder to engage than participants in other service use categories. Only two participants initiated specialist mental health treatment during the trial, and hence category 3.C had low potential for interview recruitment.

### 3.1 Objective 1: motivations for trial participation and NEON intervention usage

#### 3.1.1 Extrinsic motivations

Receiving a payment voucher was a primary motivation for trial participation for some low users in the NEON Trial. Patrick stated:

*“The reason why I used it was for the rewards you get, the voucher. I am being honest you know. Because I just need the voucher to buy things with, yeah. And also because of the issues I have got as well helps obviously.” (Patrick, NEON, Low user)*

Some low users described having no further interaction with the NEON web-application once they had claimed their voucher, including never using the NEON Intervention.

The NEON Intervention was used by some only in preparation for their process evaluation interview, motivated by knowledge that they would be questioned about their NEON Intervention usage. When reflecting on the spreadsheet describing her usage, Rose expressed:

*“Yeah, I didn’t use it that much, that [increase in use] was because I knew this interview was coming up.” (Rose, NEON, Low user)*

Usage after the primary endpoint was not accounted for in the definition of groups used in the purposive sampling strategy, but was reflected in the usage summary spreadsheet shown to participants, and hence has not influenced the usage category participants were placed into.

#### 3.1.2 Intrinsic motivations

High users were typically intrinsically motivated, i.e., they wanted to use the NEON Intervention for their own sake, with the intention of reaching their personal goals. We identified three specific intrinsic motivations described by participants.

##### 3.1.2.1 To explore the potential of an alternative or additional recovery approach

Some engaged with the NEON Intervention to explore its potential as an **alternative** to statutory National Health Service (NHS) Mental health services in England, which often have long waiting lists and capacity limitations. Some engaged to explore its potential as alternative to pharmaceutical treatment, especially for those with no desire to use medication long-term. For example:

*“I had I felt, I felt like I tried everything available to me and I was sort of waiting on NHS services, which obviously take forever and I'm kind of willing to try anything at that stage, I think like most people, I don't want to have to take medication for the rest of my life and I'm very keen on non-pharmaceutical options and this seemed quite interesting.” (Serena, NEON, High user)*

Some participants used the NEON Intervention as an alternative to mental health services that they had no access to, despite actively pursuing them:

*“Well, basically I can't get any mental health support in my area because I am diagnosed with a personality disorder … because I have got a formal diagnosis, I am refused any help in my area because … they don't have anybody qualified to deal with my complexity of issues. I have tried alternative routes to try and get help. In [location redacted] I had a very good support system, and they were very helpful and luckily, I did some DBT therapy but when I moved to [location redacted] it is a completely different system, they left me with no support whatsoever. So that's what motivated me really to use NEON, to give me the help I can’t get elsewhere right now.” (Zendaya, NEON, High user)*

This alternative status meant that NEON Intervention usage could be influenced by changing patterns of health service availability. For example, usage could be lower if alternative support became available, and greater as it became less available:

*“The only thing with NEON is when I have got my care co-ordinator, I have got my therapist, I have got my employment support and I've got all my other things that I get from the EIS (Early Intervention Service), I used NEON less then because I've got so much support from them but I probably will use it more when I don't have any of their services in December and I'll use it again next year because I won't have any support then.” (Hattie, NEON, High user)*

Some participants described a motivation to explore the NEON Intervention as an **addition** to a pre-existing set of mental health strategies:

*“I wanted to see if this tool would add anything different to what I already do for my mental health, but I’ve already got a good routine going, so maybe this tool would’ve been more years ago, right at the start of it all.” (Dorian, NEON-O, High user)*

##### 3.1.2.2 To search for answers about mental health

The NEON Intervention was described as a “safe place” by some participants who stated that they felt it was a secure platform to search for answers regarding their mental health experiences. This sense of security enabled participants to safely meet their other intrinsic motivations such as interest in recovery narratives and learning different forms of recovery. Users also appreciated that NEON Intervention narrators were open about their struggles and the reality of mental health.

*“Obviously I have a history of mental illness so it was always a case of anything I can do to help and yes it was a really interesting, especially reading stories, other people's recovery stories and yes in a way as well I wasn't expecting it to make that much of a difference but when it did oh there's other people like me and I was really interested to read and learn from that point of view and NEON was a safe place to do that as well.” (Paul, NEON-O, High user)*

##### 3.1.2.3 Due to working as a mental health professional

An unanticipated finding was that some trial participants were professionals who worked in the field of mental health (including healthcare workers and researchers), whose primary stated motivation to register for the NEON trials and access recovery narratives was to gain knowledge that aided their work around mental health conditions, rather than for the personal mental health benefits of narrative access. Erin described how her experiences with the NEON Intervention influenced her working practices as a peer support worker:

*“… I did do a searching using psychosis and then found appropriate stories just to kind of support the work that I was doing, yes, I did use it for that and it did work in that respect quite well.” (Erin, NEON, High user)*

[The NEON Intervention does not allow keyword searches and hence in this example we infer that Erin was inspired by NEON Intervention usage to search for published recovery narratives].

Florence described looking for insights arising due to narratives being under the control of narrators:

*“So, I come under the job titles like service user researcher, so everyone in our team has their own personal experience and mental health problems, I listen to, and I talked to people who have mental health problems in my work all the time. And, you know, that's more kind of conversation. So, I thought this would be a really good opportunity to see all the other ways that people kind of…because there's a difference between, you know, having a conversation, and then someone writing something that sort of might be anonymous. You know, the people that are kind of, I suppose it's, maybe they've written them in a bit more of a safe way. So, people could maybe be more honest, was what I was anticipating or maybe hoping for some good insight or for them to be helpful.” (Florence, NEON-O, High user)*

Whilst for these and other participants, motivation to participate was grounded in their profession, we have verified through inspection of transcripts that all participants who were motivated in this way were also legitimate trial participants, in that they consistently met inclusion criteria, including having personal experience of mental health problems.

### 3.2 Objective 2: appropriation of the NEON intervention

Some participants described how they consciously integrated the NEON Intervention into their everyday lives, developing their own approach to working with it which provided benefits for them. For some high users, NEON Intervention usage was an almost daily occurrence; this is reflected in usage logs demonstrating that some users accessed hundreds of unique narratives through the NEON Intervention. Descriptions of successful appropriation through thoughtful use were particularly likely to be provided by high users. The conscious nature of these processes is evident in emergent participant usage of the term “tool” to describe the NEON Intervention, usage of which demonstrates that some participants were actively thinking about the properties of the NEON Intervention and how these might fit into their lives. Descriptions of appropriation were present across a broad range of interviews with both high and low users, e.g.,

*“I really believe in therapies other than medication, I mean I also do take medication, but I believe in talking therapies and all that sort of thing, so I'd heard about these recovery stories and I thought it maybe the tool for me and I was really poorly at the time, so I really wanted to do it for myself, so that's why I took part.” (Trish, NEON, High user)*

Participants also described a broad range of outcomes of NEON Intervention use which were enabled by successful appropriation. These were mostly congruent with outcomes identified in the NEON Impact Model (20), a validation of which will be reported elsewhere. Below, we describe the three forms of appropriation which were most strongly evident in interviews.

#### 3.2.1 Tool for distraction

The NEON Intervention was appropriated as a source of distraction by some, for example as a distraction from negative internal thoughts, through being immersed in the narration of others.

*“Even though I kind of like I throw myself into the world of stories, and that does distract me, usually from me. I never use it as a kind of coach, but I think their words do kind of distract me even on a subconscious level which is brilliant. And so that source of distraction is important whatever you do.” (Kieran, NEON-O, High user)*

*“I feel like mental health services have a role like in my life have a massive role. But like there's stuff outside of services that also impacts on like how you are. So, I was like, reading people's stories or looking at artwork that they've made which was helpful for me. So, these stories became something that I could add to my daily arsenal of like, Oh, if it's a rubbish day, or like, if I need distraction …” (Tia, NEON, High user)*

Grace indicated that for her, the happier narratives were the ones accessed more for distraction:

*“There's sometimes when you can get really, really to a point when you’re at your lowest. When I’m there I just keep myself in that low mindset, which obviously, it could be quite counterproductive. It's just, it's trying to crack into that. And get yourself to do something. The stories helped distract me, but I need to say that it was more of the happy ones. I think I used it a lot for happy distractions.” (Grace, NEON, High user)*

#### 3.2.2 Tool for emotional boost

Some participants reported turning to the NEON Intervention when they needed to boost their mood.

*“These stories were a kind of quick pick me up almost that I could use daily.” (Tia, NEON, High user)*

*“So, I kind of thought okay this is what obviously maybe give me a bit of inspiration to uplift me when I needed it. You know if people have recovered and have gone to extra learning and be able to do full time jobs and it was just kind of give me a little glimmer of hope that there's something beyond this point and when you're at your lowest point, you need something to bring you out of that, give you hope and you think you're not alone. I’d go on NEON when I needed that uplift.” (Hattie, NEON, High user)*

#### 3.2.3 Tool for sustaining the perception of a support network

Some participants described perceiving NEON Collection narratives as a support network that enabled an indirect sense of connection with others, and which made them feel a part of a supportive community, despite having no direct contact with NEON Intervention narrators:

*“It's a bit like a bit of inspiration and a bit of anonymous support in a round-about way, you can't help but be inspired, I mean there's some stories on there you think how did they get out of it or how do you get that low down in recovery (…) it's a support network. As I say, it doesn't matter which story you're reading, even the ones that I couldn't relate to, they've all been inspiring as a support network.” (Matty, NEON, High user)*

This was congruent with the outcome of “connectedness” described in the NEON impact model, and elucidated in other work (23), and was a particularly helpful form of impact for individuals that felt isolated from friends and family.

In common with other participants, and as well as using NEON as a tool for emotional boost, Matty described turning to the NEON Intervention due to his perception of it as a support network, and due to help that came from enhancing this perception:

*“… it gets to the point that it book marked on my phone, the website, then you start thinking I'll just go back and read a story here, see how someone else dealt with it and it ends up a sort of staple and you start using it as a resource for recovery, so when I've been getting more and more down I've found myself instead of listening to a podcast go back and just reading through some NEON stories, there were various other resources I used as well but as my mood has gone down I've used NEON more as a resource to get myself back up again and as a little support network, even though you don't know the people who've written the stories, a little support network and you can relate to it. NEON has grown into this fabulous resource, I mean my own personal circumstances have changed this year regarding my mental health but it's nice to have a new resource, almost like a peer support, although you don't know who these people are it's like peer support, it's just been a fantastic tool to have and I feel I've just been lucky that I've stumbled across NEON at the same time as I needed them” (Matty, NEON, High user)*

### 3.3 Objective 3: perceptions of the NEON collection

Participants discussed their perceptions of the NEON Collection in substantial detail. We identified the following findings as being of particular salience to DHIs integrating narrative collections.

#### 3.3.1 Disengagement after early contact

The first few narrative(s) that participants were recommended or selected themselves influenced their engagement with the NEON Intervention for some. Some low users stopped using the NEON Intervention when the initial narratives they encountered were perceived as not relevant:

*“I didn’t read more than three, maybe four. To be honest they just weren’t relevant to me, kind of a waste. I just stopped using it after that. There was no point, do you know what I mean?” (Yara, NEON-O, Low user)*

Not finding the right content could lead to a negative evaluation of the NEON Collection as a whole (even if that content was available, but the participant had not succeeded in locating it):

*“It's kind of only really reflecting on it now, that I realised that that might have been why I didn't really use it much. Because I didn't find the content that I needed to begin with, which maybe made me feel like it wasn't helpful.” (Alice, NEON, Low user)*

These experiences were not useful; some high users described persisting with the NEON Collection, even if they did not immediately find helpful narratives.

#### 3.3.2 The desired narrative

Many participants felt they could articulate characteristics of recovery narratives that might most benefit them, and desired to find narratives with these characteristics in the NEON Collection. They frequently felt more engaged with the NEON Intervention if they found narratives that matched their ideal, and less engaged if they failed or found it difficult to find such a narrative. Hence being able to find a narrative with their desired characteristics influenced NEON Intervention engagement for those participants who felt they could articulate those characteristics. In Table 2, we synthesise the characteristics of desired narratives described by participants.

**Table 2:** Characteristics of narratives considered ideal by study participants.

Narratives with the potential for impact	Narratives with the potential to shift the participant's understanding of their mental health experiences
	Helpful narratives [nb. Participants frequently indicated that they wanted to find narratives with the potential to help them, without being specific about what this mean]
Narrative form	Narratives that avoid formulaic structures
	Positive narratives
	Hopeful narrative
	Reflective narratives
	Brave narratives
	Authentic narratives
Narrative content	Narratives about isolation
	Physical health narratives
	Religious narratives
	Music-based narratives
	Up to date narratives
	Narratives describing a broad range of mental health diagnoses
	Advice-based narratives
	Experience-focused narratives [that avoided giving advice]

Some forms of desired narrative content presented in Table 2 were identified from transcript fragments where participants described content that they perceived as missing from the NEON Collection, but would ideally like to have found. For example, the NEON trials were open during a period that roughly coincided with the COVID-19 pandemic, and some participants desired to find narratives describing recovery from mental health experiences relating to events occurring in the pandemic, or due to the sustained period of isolation encountered during the pandemic (which were not present in the NEON Collection). Some participants desired narratives describing recovery experiences from a broader range of mental health diagnoses than were explicitly available—many NEON Collection narratives do not explicitly use diagnostic labels when describing mental health experiences, and hence participants looking for narratives discussing less-common diagnoses may not have found them. Most generally, we can conclude that there is no narrative that would be perceived as ideal for all participants, as e.g., advice-based narratives and experience-focused narratives were coded as mutually exclusive categories due to how they were described.

#### 3.3.3 Characteristics of narratives perceived as authentic

The perceived authenticity of narrator and narrative was reported by some as a central factor on which they decided whether to continue interacting with a narrative.

*“But as long as it's authentic, the worst thing would be to force all the stories into saying that narrative of hope. It would then start to feel contrived and not real. So, like I say, although maybe a little bit disappointing at the time the video story didn't really take me anywhere, it still felt it gave me trust, that these were genuine stories, and not sort of fake stories told for a purpose, which I wouldn't enjoy at all.” (Rob, NEON, Low user)*

For some users, an anonymous narrator aided perceptions of authenticity, since being anonymous in a narrative could be perceived as a human response to mental health stigma or perhaps a mechanism enabling honesty that may not otherwise have been possible:

*“I really liked the stories where the author had no name, you didn't know who they are, they were just that bit more believable.” (Angela, NEON, High user)*

We examined all discussions of authenticity in transcripts, and identified characteristics of narratives that were more likely to lead to perceptions of authenticity. These were:

• Acknowledgement of difficulty: the narrator acknowledges the difficult reality of mental health experiences
• Realism: The narrative appears to describe real world experiences
• Shared experience: The mental health experiences of the narrator are similar to those of the participant [enabling the participant to relate to those experiences]

The final point suggests that perceptions of authenticity (or not) can arise as an interplay between characteristics of the narrative and of the recipient, and hence that perceived authenticity is not simply a feature of a narrative alone.

#### 3.3.4 Scale and diversity of the NEON collection

For most participants, the substantial scale and diversity of narratives in the NEON Collection supported people to remain interested and engaged in using the NEON Intervention.

*“I found it very interesting, I found that the stories were very, some of them they were powerful and coming from a lot of insight of the lived experience and very so it was quite a big variety of some experiences, so it's just very, very every sort of diagnosis and so yes I found it very well done, kept me interested, and it was very well think of and very easy to follow and yes and powerful as well yes.” (Katlyn, NEON, High user)*

Some participants who were high users greatly appreciated the large scale of the NEON Collection. For example, always being able to access new narratives kept a high user [178 narratives accessed] engaged despite experiencing ADHD:

*“… I had a really late diagnosis of ADHD which explained a lot of things and now it's about really making the most of what I'm good at rather than kicking myself because I can't do Excel spreadsheets because it's not that I don't try hard enough, I just don't quite have the skills or the neuron connections to do it like other people. So yes, NEON's good, I love the fact that despite all efforts I haven't been sent a story twice, that's pretty cool and kept me intrigued to read and explore more.” (Stefanie, NEON, High user)*

High users also reported that they appreciated there being sufficient narratives they could access in the NEON Intervention regularly.

The scale of the NEON Collection was found to be overwhelming for some due to the magnitude of narratives available, and this affected engagement:

*“There were so many stories that I sometimes switched off.” (Angela, NEON, High user)*

Erin found that NEON Collection use led her to access narratives outside of the NEON Intervention, and that the scale of what was available outside of the NEON Intervention could be overwhelming.

*“I think there's other stories on there that might have a similar impact on me that I've not found yet, not discovered that collection of stories so it's kind of good that I would still have ongoing access so that I can find out a little bit more about things that I might have missed but also I suppose I can see why it's overwhelming because I've set up the thing where I carry on researching even more and look into other stuff so I suppose it kind of opens up even more avenues but those are ones that I've chosen to go down myself, rather than it all being on NEON.” (Erin, NEON, High user)*

Finally, having a variety of narrative formats was perceived as unanimously beneficial, for example as a mechanism for accommodating opposing preferences by different participants.

*“Just to know that there's a story here for you, it's kind of a thing where there is a good variety of stories. I think format is important for people. You know, I don't like listening to audiobooks, whereas people I know absolutely love it. And that's how they get their books. We've got images, videos, so, it's sort of a case of what you want.” (Rob, NEON, Low user)*

## 4 Discussion

### 4.1 Principle findings

Our findings have demonstrated that the NEON Intervention was successfully appropriated by some participants, and suggest that appropriation is a factor in high use. Some engagement (or the lack of it) was explained by participants' perceptions of the NEON Collection that underpins the NEON Intervention. Engagement was typically enhanced when people found narratives that they perceived as authentic, or narratives with characteristics that matched those that they desired to find. For some participants, failing to find relevant narratives early on contributed to low engagement, and could contribute to a perception that the NEON Collection as a whole lacked relevance for them, even though this might not have been correct. The diversity of narrative formats contained in the NEON Collection was universally perceived as positive, and the large scale and diversity of content was perceived as positive by most. A minority of participants found the scale of the NEON Collection overwhelming, which contributed to disengagement for some. Engaging with narratives in the NEON Intervention could lead to participants searching for recovery narratives outside of the NEON Intervention, but in turn the scale of what is publicly available could feel overwhelming. Some participants found that accessing the NEON Collection provided a perception of having a distributed social support network despite there being no mechanism to directly engage with narrators or other users. Narrative collections such as the NEON Collection may be a mechanism for creating a beneficial perception of *common humanity*, ie. a recognition that struggle is a common human experience across all societies (43).

### 4.2 Relationship to prior work

Some trial participants perceived anonymous narratives as being authentic, due to a belief that a narrator would choose to be anonymous because of anticipated stigma about mental health problems. However, our own work suggests that anonymity can be enforced on narrators by curators of narrative collections who fear narrator harm through narrator identifiability (2). Since The NEON Collection integrates narratives from more than thirty existing collections with varying curatorial processes, then it is possible that this anonymity, and hence these perceptions of authenticity, were due to curator rather than narrator choice.

In parallel, Winstone et al. have examined how people who self-harm evaluate lived experience narratives. They found that perceiving a narrative as authentic can contribute to help-seeking by a recipient, and that narrative authenticity can be promoted by factors such as presenting realistic representations of recovery as a non-linear process (include setbacks or relapses), and honesty about self-harm as a coping mechanism. They recognise that presenting more “extreme” accounts of crisis might contribute to perceptions of authenticity whilst raising a danger of triggering self-harm in participants (44). This is in keeping with our own systematic review on the impact on recipients of recovery narratives, which identified that access to recovery narratives describing eating disorder experiences might encourage disordered eating in susceptible participants (22). Collectively, these insights point to narrative authenticity as both an opportunity and a responsibility challenge for narrative intervention developers, who may wish to minimise potential harms, while maximising both narrative impact and user safety.

Work to understand perceptions of authenticity may be informed by a wider body of related work on the credibility of health information (45) which demonstrates that sources perceived as having higher credibility are also perceived as more useful to individuals (45). The collation of narratives for narrative interventions may be supported by artefacts such as production guides, which can accumulate knowledge on what makes a narrative feel authentic. A video recovery narrative production guide has been co-produced and published by the KLIFAD study, which has examined the impact of alcohol misuse recovery narratives on people misusing alcohol. The KLIFAD production guide contains content enabling videographers to support participants in expressing narratives likely to be perceived as authentic, derived from an existing theory base (46). This guide was used to produce a collection of video recovery narratives for use in a feasibility trial (47). Since narrative diversity was mostly perceived as beneficial, then people curating collections may benefit from guidance on how to assess and plan for diversity. Kotera et al. have proposed diversity and inclusivity metrics for recovery narratives. These are broadly applicable beyond mental health (48), and are also well-aligned with responsible innovation practices (49) which are emerging as critically-important issues in healthcare research and development.

### 4.3 Strengths and limitations

Strengths include the use of a purposive sampling strategy that enabled access to a broad range of NEON Intervention experiences, from those that involved regular use, to those that involved no use. Whilst we sought for variation on service use history, we were limited by being unable to recruit participants who had never used any form of statutory mental health service, who were hard to engage. Future studies may consider new tactics to reach people in this category, such as enhanced payments, or prospective invitations to interview at the point of trial registration. Whilst we made a deliberate choice to only interview participants after the primary endpoint, so as not to influence quantitative outcome assessment, our interviews were reliant on participant memory. Our findings may have been influenced by the impact of the COVID-19 pandemic, including government-imposed restrictions on socialisation and mobility, which coincided with much of the trial recruitment and follow-up period.

### 4.4 Recommendations

Drawing on our findings and discussion, we propose the following recommendations to promote responsible innovation practices aimed at intervention developers and implementers.

(1) DHIs using digital media collections should make the scale, diversity, and inclusivity of collections clearly visible to users at first contact and beyond, and should provide access mechanisms that avoid users feeling overwhelmed.

If users can be rapidly deterred by perceiving a digital media collection as limited, then attempts to communicate the scale, diversity, and inclusivity of a media collection may enhance engagement. To support users at risk of feeling overwhelmed, more limited views of a collection should be provided. One tactic would be to provide smaller sub-collections of digital media items, for example a recommended set of ten narratives describing recovery after depression.

(2) Intervention developers should provide delivery mechanisms that afford appropriation

For example, since we found that some users accessed narratives on an almost daily basis, then a digital service providing a recommendation per day might afford appropriation by people who find benefits in that particular pattern of use.

(3) Curators of narrative collections should enable their contributors to produce authentic narratives.

This may involve creating resources such as production guides setting a clear expectation that authentic narratives are acceptable, and that narrators do not need to hide their experiences (whilst setting boundaries on experience representations that may cause harm to others, such as graphic descriptions of self-harm).

(4) Intervention developers should enable learning by legitimate users who are also healthcare professionals

This may provide an alternative route to impact from the intervention, for example by healthcare professionals using knowledge gained from recovery narratives to improve their own practice, or enabling healthcare professionals to recommend narratives from a collection to their clients.

(5) Web-based delivery of recovery narratives might be used to tackle loneliness across different health conditions due to their capacity to create a perception of having a social network

Implementation work might target who could benefit from feeling socially connected, which in turn may improve mental health (50–52).

## 5 Conclusions

Through our process evaluation of the NEON Trial and NEON-O Trial, we have developed knowledge on motivations for trial registration and NEON Intervention usage, forms of appropriation, and perceptions of our collection of mental health recovery narratives, and used to our findings to identify specific recommendations for developers of interventions making use of digital narratives. In considering how our intervention was appropriated, we have described three specific reasons for appropriation that were presented by our participants. Given that most use of digital healthcare technologies will need to take place as part of normal daily routines, and will have to sit well with those routines, then we propose that a research focus on understanding appropriation is important (though of course technologies can still be appropriated by people who have been separated from daily routines, such as people who have been hospitalised). In keeping with its study outside of healthcare, the study of appropriative processes for digital healthcare technologies may require socially oriented methods such as ethnomethodology, to provide direct observation of appropriative processes at work.

## Data Availability

The raw data supporting the conclusions of this article will be made available by the authors, without undue reservation.
