# Supplementary material for: Perception and appropriation of a web-based recovery narratives intervention: qualitative interview study
Source: Front Digit Health. 2024 Feb 14;6:1297935. doi: 10.3389/fdgth.2024.1297935 (PMC10899698; doi:10.3389/fdgth.2024.1297935)
Supplement: Supplementary file 1 [file Datasheet1.docx]

Appendix 1 – Forms used to collect service use information

The following two forms were used to collect information about participants in the NEON Trial and the NEON-O Trial. This appendix only includes forms that collected information on participant health service use. The full set of baseline forms has been published as supplementary information to our trial protocol: <https://doi.org/10.1186/s13063-020-04428-6>.

For each form we have summarised:

- The title and subtitle that was visible to participants
- Any necessary instructions on how fill out the form
- The items that were included, and their legitimate values

# Demographics form

**Title**: Form 1/10 – information about you

**Subtitle**: This information will help us understand who is taking part in our trials

**Items**

1. What is your age in years? [Response options: integer from 18 upwards, no upper limit]
2. What best describes your gender? [Response options: Female, Male, Other]
3. What best describes your ethnicity?

Response options: Items in italic can be selected, other items provide structure. Only one item to be selected from the following.

White
 *British*
 *Irish*
 *Gypsy or Irish Traveller*
 *Any other White background*
Mixed / Multiple ethnic groups
 *White and Black Caribbean
 White and Black African
 White and Asian
 Any other Mixed / Multiple ethnic background*
Asian / Asian British
 *Indian
 Pakistani
 Bangladeshi
 Chinese
 Any other Asian background*Black / African / Caribbean / Black British
 *African
 Caribbean
 Any other Black / African / Caribbean background*Other ethnic group
 *Arab
 Any other ethnic group*

1. What region of England do you currently live in?
   [Response options (one only): East of England, London, Midlands, North East and Yorkshire, North West, South East, South West]
2. What best describes your highest qualification? [Response options (one only): No qualifications, O-levels/GCSE or equivalent, A-levels/AS-levels/NVQ or equivalent, Degree-level qualification, Higher degree-level qualification]
3. Have you ever (including currently) used primary care mental health services, e.g. had support or medication prescribed by your GP for mental health issues, seen a GP practice counsellor, or used the Increasing Access to Psychological Therapies (IAPT) programme?
   [Response options (one only): Yes, No.]
4. Have you ever (including currently) used specialist mental healthcare services, e.g. a community mental health team, mental health in-patient ward?
   [Response options (one only): Yes, No.]
5. [NEON Trial only] Which of the following best describes the current contact you have with the NHS about your experiences of psychosis
   [Response options (one only): No contact with any NHS service, Contact with my GP, Contact with primary care counsellor, Contact with Improving Access to Psychological Therapies (IAPT), Contact with a specialist community mental health team, Currently a mental health in-patient in hospital]
6. Which of the following best describe the main mental health problem you have experienced in the last month? .
   [Response options (one only): I don’t want to say, I did not experience mental health problems, Developmental disorder such as learning disability, Eating disorder such as anorexia or bulimia, Mood disorder such as depression, anxiety or bipolar, Personality disorder such as borderline personality disorder, Schizophrenia or other psychosis such as schizo-affective disorder or delusional disorder, Stress-related disorders such as PTSD or OCD, Substance-related disorder such as alcohol or drug mis-use]

If any options other than “I did not experience mental health problems” or “I don’t want to say” are selected for question 9

1. Which of the following best describe how you are thinking about your recovery from mental health problems? These terms have been taken from a research model.
   [Response options (one only): I don’t want to say, Not yet thinking about recovery, Working on recovery, Living beyond disability]

# Health economics measure of service use: Abridged CSRI

**Title**: Form 8/10 – Your use of health services

**Subtitle**: This information will help us to calculate how cost-effective NEON is.

**Items** *Follow up questions in italics*

| In the last [**6\|12**] **months** how many nights have you stayed in hospital because of… | |
| --- | --- |
| **…**mental health problems? | BOX1 [0,1,2, ...] |
| …any other reason (e.g. physical health problems)? | BOX2 [0,1,2, …] |
| *[If BOX1 or BOX2 has non-zero response and if the form is being completed as part of the 52 week follow-up]* |  |
| *Did taking part in this trial contribute to you going into hospital or staying longer than expected?* | BOX1-2a [yes, unsure no] |
|  |  |
| *[If response to BOX1-2a is “yes” or “unsure”] How might the trial have contributed?* | BOX1-2b  [multi line free text] |
| *When did you go into hospital?* | BOX1-2c [date selector] |
| *Which hospital were you admitted to?* | BOX1-2d  [multiline free text] |
| In the last [**6\|12] months** how many visits have you made to… | |
| Accident and Emergency (A&E) department or a Minor Injuries Unit? | BOX3 [0,1,2, ...] |
| *[If BOX3 has non-zero response and if the form is being completed as part of the 52 week follow up]* |  |
| *Did taking part in this trial contribute to you going to A&E / Minor Injuries Unit?* | BOX3a [yes, unsure, no] |
|  |  |
| *[If response to BOX3a is “yes” or unsure] How might the trial have contributed?* | BOX3b [free text] |
| *When did you go to the A&E / Minor Injuries Unit?* | BOX3c [date selector] |
| *Which A&E / Minor Injuries Unit did you go to?* | BOX3d  [multiline free text] |
| General practitioner / family doctor | BOX4 [0,1,2, ...] |
| Community nurse, occupational therapist, primary care counsellor, IAPT therapist OR family therapist | BOX5 [0,1,2, ...] |
| Psychologist | BOX6 [0,1,2, ...] |
| Psychiatrist | BOX7 [0,1,2, ...] |
| Day care service | BOX8 [0,1,2, ...] |
| Boxes 1-8: Defaults to no value. Valid response is an integer from 0 to 183 (6 months retrospective) or 0 to 365 (12 months retrospective)  BOX1-2a and BOX3a: Defaults to no value. User must enter one of the available response options.  BOX1-2b and BOX3b: Some text must be supplied  BOX1-2c and BOX3c: Date must be supplied  BOX1-2d and BOX3d: Some text must be supplied | |
| How many weeks have you worked in the last [6\|12] months? | BOX9 [0,1,2, ... 52] |
| Which of the following best describes your current employment situation? *(One option to be ticked. Follow ups in italics)* | BOX10 |
| Option 1: In paid employment |  |
| *How many hours per week do you typically work in all your paid jobs?* | *BOX10a [1,2, …]* |
| *What is your main job?* | *BOX10b [Options below]* |
| Option 2: Unemployed and looking for work |  |
| *When were you last employed?* | *BOX10a [Date entry]* |
| *What was your most recent job?* | *BOX10b [Options below]* |
| Option 3: At home and not looking for work (e.g. housewife/husband) |  |
| Option 4: Unable to work due to illness |  |
| *When were you last employed?* | *BOX10a [Date entry]* |
| *What was your most recent job?* | *BOX10b [Options below]* |
| Option 5: Unable to work for another reason (e.g. caring for someone) |  |
| *When were you last employed?* | *BOX10a [Date entry]* |
| *What was your most recent job?* | *BOX10b [Options below]* |
| Option 6: Retired |  |

Response options for BOX10b: Manager/administrator, Professional (e.g. health, teaching, legal), Associate professional (e.g. technical, nursing), Clerical worker/secretary, Skilled labourer (e.g. building, electrical etc), Services/sales (e.g. retail), Factory worker, Other.
