# Supplementary material for: Perception and appropriation of a web-based recovery narratives intervention: qualitative interview study
Source: Front Digit Health. 2024 Feb 14;6:1297935. doi: 10.3389/fdgth.2024.1297935 (PMC10899698; doi:10.3389/fdgth.2024.1297935)
Supplement: Supplementary file 2 [file Datasheet2.docx]

**Appendix 2: NEON Trial and NEON-O Trial Process Evaluation Interview Topic Guide**

Contents

[Consent & Start of Interview (UP TO 5 MINS) 2](#_Toc76625111)

[Initial thoughts & Accessibility (Up to 10 MINS) 3](#_Toc76625112)

[Context (Up to 5 MINS) 4](#_Toc76625113)

[Patterns of usage (Up to 5 MINS) 4](#_Toc76625114)

[Content Relevance (Up to 5 MINS) 5](#_Toc76625115)

[Individual narrative VS. The collection (Up to 10 MINS) 5](#_Toc76625116)

[Harm & Safety (Up to 5 MINS) 6](#_Toc76625117)

[Change: Positive or negative impacts (Up to 10 MINS) 7](#_Toc76625118)

[Interview ending (5 MINS) 7](#_Toc76625119)

[Process Evaluation Interview Distress Protocol 8](#_Toc76625120)

[Interview procedure: Serious Adverse Events Reporting 9](#_Toc76625121)

[Interview procedure: participant is a risk to self 11](#_Toc76625122)

[Interview procedure: participant is a risk to others 11](#_Toc76625123)

[Interview procedure: concerns around capacity 12](#_Toc76625124)

Topics are shown as bullet points, with example follow-up and probing questions which will be used to elaborate topics of interest. This topic guide assumes that interviews will be conducted using Microsoft Teams as this is the default interviewing method unless participants prefer a phone call. If it’s a phone call interview the researcher should modify the script accordingly.

# Consent & Start of Interview (UP TO 5 MINS)

**Example opening of interview:**

Before taking participant’s consent:

Thank you very much for being willing to take part in this interview. This interview will involve you talking about your experiences of using the NEON online intervention, and your thoughts on how useful it was for you. As you know we’ll be asking about how frequently you used NEON based on the recorded data we have of your usage. Please feel free to be completely honest with us with what you liked and didn’t like about NEON. Your true experience and opinion of it will help us improve NEON in the future, so we really appreciate it. You can have the option to turn off your camera if that makes you feel more comfortable. Also, if you would prefer me to have my video off that wouldn’t be a problem (interviewer to act according to the participant’s preferences).

Just a reminder that anything you tell me during the next hour is confidential to myself and the small team of researcher’s developing NEON. The only situation where we’d consider passing things on to others is if we felt you were at risk of harm or others were at risk of harm, and we’d always try to work out with you what the next steps would be. Are you still happy to take part? (If participant answers No= stop interview and thank them), if participant answers yes= proceed with taking verbal consent).

(Start audio/teams video recorder). Can you please say, “I consent to take part in this interview and for it be audio/video recorded?” (Wait for participant to repeat)

After taking consent: Great! I just want you to know that your comfort is really important to us, and we want you to be as comfortable as possible during the interview so can take a break at any time, so just let me know whenever you want one. The interview’s time might go a bit beyond an hour if we do take a break, would that be okay with you? (Wait for answer) As a research team we completely respect and appreciate everything you’re sharing with us. So, let’s begin!

**Questions* *to be adapted by each interviewer to suit personal preference.*

*The overall aim of the process evaluation is to characterise the participant experience of taking part in the NEON and NEON-O trials. This aim is pursued throughout the questions in the topic guide.*

# Initial thoughts & Accessibility (Up to 10 MINS)

1. What did you think of NEON in general?

*(Prompts- only if narrator comes to a stand-still: Any general thoughts about your experience using the intervention.)*

1. What was your motivation to take part?
2. What were your expectations of NEON before using NEON?

*(Prompt: Did it meet your expectations?)*

1. How did you find NEON visually?

*(Prompts: How was the layout, font size, length of text on a page?)*

1. When you first started using NEON, how did you find the features available?

*(Prompt: Were there any features that you found difficult or confusing? Are there any features that you still find difficult or confusing? How did you find the Matching of stories feature?)*

***Features*** *include Bookmark button, Matching algorithm, Self-management techniques section, get me out of here button, random button, Categories selection etc.*

1. Do you have any previous experience reading or watching similar stories (i.e., stories outside of NEON)?

# Patterns of usage (Up to 5 MINS)

1. It’s always been up to you how much you used NEON, but we’re interested in things that might get in the way of you using. (Personalize according to participant’s usage data for the main question) e.g. So, between January and March you used NEON very frequently, could you tell me a little bit more about that if that’s okay?

*(Prompt: It’s been an unusual past year; did that impact your usage at all?)*

# Were there any specific circumstances/ situations that made you use the intervention more?

# *(Prompt: Can you tell me more about that? What was the experience like for you?)*

# Content Relevance (Up to 5 MINS)

1. Did you use the matching function on NEON? [If yes] How do you feel about the story matches generated by NEON?

*(Prompt: Were they good or bad? Do you have any concerns about the matching process?)*

1. How relatable were the stories you interacted with?
2. How can we improve the range of stories available?

*(Prompts: What do you think about the stories that were recommended to you? How relevant were they for you? Did you relate to them? How?)*

1. *Thinking back to the stories you read on NEON, which were your favourite?*

*(Prompt: What about that story in particular stood out to you? Why did you like it?)*

1. *Do you have any stories that were your least favourite story?*

*(Prompt: What about the story made it unlikeable?)*

# Individual narrative VS. the collection (Up to 10 MINS)

1. What do you think of the NEON collection of stories as a whole?

*(Prompts: How diverse do you think the NEON collection is? Do you think there is anything missing from the collection of stories?)*

1. How was it having access to such a wide range of stories? (If the participant refers to the collection being diverse)
2. When you found a story you liked, what did you do after that?

*(Prompt: Did you read the single story that impacted you over again? Or access similar ones in the collection?)*

1. What impact did the stories have on you?

*(Prompt: What is it about the story that attracted you the most? Can you think of a story or stories that were particularly impactful for you, positively or negatively?)*

1. Could anything be done to have made experiencing the stories better?

# Harm & Safety (Up to 5 MINS)

1. Did you feel distressed at any point whilst using NEON?
2. (If yes) What caused you to be distressed?
3. How did you manage your distress?

*(Prompt: Did you use the “I’m upset” page? Did you specify a self-management strategy through the about me page? Did you use them? How well did it work?*

*What could be added to the NEON Intervention to make it safer?*

*How did you find the content warnings before stories?*

*Did you find the option of blocking a story/ collection useful?*

*Did you use the “Get me out of here” button at any point? [If yes] How did you find it?)*

# Change: Positive or negative impacts (Up to 10 MINS)

1. Have you noticed any changes to your thoughts, feelings, or behaviours as a result of the stories you accessed on NEON? Can you say how the story led to the change?

*(Prompt: Have stories been helpful or harmful? If so, which? Why*? Did you feel any connection to the stories you read? If so, how? *How did the stories you read make you feel?)*

1. If no impact: What could have been done to make NEON more impactful for you? (Also skip question 21 & 22)
2. What does recovery mean to you?
3. Do you feel like the stories changed your day-to-day life in any way? If so, how?

*(Prompt: Did using NEON impact at all how you view or share your own story? Have you looked at any recovery stories outside of NEON?)*

1. Looking at the past year, have you experienced any changes to how you view yourself since engaging with the NEON intervention?

*(Prompt: Did the NEON intervention/stories that you read as part of the NEON intervention lead to changes in personal identity? Personal strength/increased support/new opportunities/development of new coping strategies/gaining of new perspectives/new emotional experience/improved relationships)*

# Interview ending (5 MINS)

Thank you very much for taking part! We hope you enjoyed the interview! If there’s any further feedback, you’d like to offer on NEON please send me an email using the NEON email address: [neon@nottingham.ac.uk](mailto:neon@nottingham.ac.uk) . Just as a thank you we’re paying you £20 and this can be either through a voucher or money paid directly to your bank account, for which we’d need to fill in a form with some of your bank details. Which would you prefer? (Agree with participant based on their preferences). Great that’s it for the interview then. Have a lovely rest of your day!!

Total estimated time of interview: 60 Minutes

# Process Evaluation Interview Distress Protocol

This distress protocol is placed in order to support both the interviewees and interviewer.

**Pre-data collection:**

The interviewer will self-assess their current life circumstance and the potential physical and psychological impact from listening to a participant’s experiences on themselves. This self-assessment will influence the number of interviews they can complete in a week. Interviewers will engage in their own self-care strategies to combat any potential emotional exhaustion.

**Data collection Stage:**

Not all interviewees may alert the researcher to them feeling distressed, therefore interviewers will need to actively be mindful. Therefore, the interviewer will observe the individual’s emotional reactions, non-verbal cues and verbal content, which may assist in assessing whether an individual is distressed or becoming distressed.

If the interviewee indicates they are experiencing a high level of stress or emotional distress or displaying distressed behaviours such as uncontrolled crying, shaking etc. the following actions will take place:

• Interviewer will acknowledge their interviewee’s emotional state and will offer immediate support by offering to take a 10-minute break. The participant will be asked to turn off their audio and camera and then re-join after the 10-minute break.

• The interviewer will also give them the opportunity to stop the interview and not continue. The interviewer will tell the participant they will always be paid for the interview, even if they do not complete it.

**Review:**

- The interviewer will ask if interviewee if they’d like to continue with the interview, if yes, then after stressing that the interviewee can take a break can again at any time, the interview will continue.
- If the interviewee no longer wants to participate in the interview, then the interviewer will thank them for taking part. The interviewer will suggest that the interviewee talk their distress over with their family, friend, or care team. If interviewee has completed parts of the interview, conduct debrief questions if they’re willing. Ask them to complete and send over reimbursement form, if they still want.

**Analysis Stage:**

• Researcher should alert transcriber prior to transcription review of potentially "challenging" or "difficult” interviews.

- - Transcriber has the option to pass on interview onto someone else if they feel the content is too distressing for them.

• Researcher has regular online scheduled debriefing sessions with a named member of the research team (Dr Stefan Rennick-Egglestone).

**Follow Up:**

- Encourage the researcher to discuss with Dr Stefan Rennick-Egglestone if he/she experiences increased distress in the hours/days following transcription.

# Interview procedure: Serious Adverse Events Reporting

A serious adverse event (SAE) as defined by the HRA is an untoward occurrence that:

1. Results in death

2. Is life threatening

3. Requires hospitalisation or prolongation of existing hospitalisation

4. Results in persistent or significant disability or incapacity

5. Consists of a congenital anomaly or birth defect; or

6. Is otherwise considered medically significant by the investigator.

All SAEs occurring up until the end of the NEON study must be reported to the Programme Steering Committee. Reporting will be by Stefan Rennick-Egglestone.

SAEs that are (1) related to the study and (2) unexpected must also be reported to the Research Ethics Committee governing the study. Reporting will be by Mike Slade.

SAEs are classed as **expected** (and hence only reported to PSC and not to REC) if they are caused by the participant:

1. Feeling disconnected from others
2. Feeling more pessimistic
3. Feeling emotionally burdened
4. Feeling inadequate
5. Experiencing the release of uncomfortable emotions
6. Engaging in harmful behaviours encountered in narratives

To determine expectedness and relatedness, then we are dependent upon the information the participant tells us. We can’t assume they will ever talk to us again, so we have to assume the interview is the only place we can ask about further information.

During the interview process, if the participant reports a SAE or something that might be an SAE, then enquire whether they have already provided details about it through one of the NEON online forms. **If they have already reported it**, then no further questioning about the SAE is needed, so thank them for reporting it, and continue with the interview.

If the participant confirms they have **not** reported the SAE already, then obtain an elaboration of the SAE from the participant, for example:

“Please tell us as much as you can, as this will help us to make sure that our intervention is as safe as it can possibly be.

Ideally, we need responses to the following five questions, but Q1-Q3 are the most important.

Q1: What happened?

Q2: In your opinion was this caused by your participation in a NEON trial?

Q3: If yes then ask: Please tell us how you think the trial might have caused this.

Q4: When did this occur? Please give us your best guess if you cannot remember accurately.

Q5: Where did this occur?

Once you’ve received responses, then you might say:

“Thanks for telling us about this. Is it ok for the interview to continue now?”

After the interview has completed:

1. summarise responses to Q1-Q5 into a document called [participant ID] Process evaluation safety report dd.mm.yy, e.g. “1001 Process evaluation safety report 21.06.21.docx”

2. place into R:\DRS-RRT-NEON-Trial\1. Safety reports\Process evaluation safety reports

3. notify **SRE only** if the safety report is clearly unrelated to the study (by email)

4. notify **SRE and MS** if the safety report may be related to the study (by email)

# Interview procedure: participant is a risk to self

If the interviewer feels the participant might be a risk to self, they should talk through with the participant the following questions:

- What they will do to stay safe?
- Is there anything that you can do to help?
- If you feel the person is at immediate risk to themselves: Call the police on 999 using a mobile phone or landline. Before doing this say the following to participant:

*“From what you’ve said I’m a bit worried that you’re at risk to yourself/ someone else and so I’d like to contact the police. Can you tell me where you are?”*

- After the interview, inform Stefan Rennick-Egglestone by email of what has happened, or if situation feels urgent call 07504 331005. Always pass on the person’s trial ID to SRE in every communication.

# Interview procedure: participant is a risk to others

If the interviewer feels the participant might be a risk to others, they should talk through with the participant the following questions:

- Who might they be a risk to?
- What form of risk might they pose?
- What might the participant do to
- If you feel the person is at immediate risk to others: Call the police on 999 using a mobile phone or landline, passing on as much information as you can about the person who they might be a risk to.
- After the interview, inform Stefan Rennick-Egglestone by email of what has happened, or if situation feels urgent call 07504 331005. Always pass on the person’s trial ID to SRE in every communication.

# Interview procedure: concerns around capacity

Capacity means the ability to use and understand information to make decisions (such as the decision to take part in an interview or to answer specific questions), and communicate any decision made.

A person lacks capacity if their mind is impaired or disturbed in some way, which means they're unable to make a decision at that time. For example, you might feel that they can’t make an informed decision to take part in the interview.

If you are concerned at any time during the interview that the individual doesn’t have capacity to give informed consent or take part in the interview, take these actions:

Ask them: Shall we stop for a second. I’ve noticed that you might be… (say what you noticed). **If they appear distressed follow the distress protocol**. After a break if participant still seems not have capacity to follow through with the interview, stop and the interview and say the following:

Tell participant “I think we should stop the interview here. Thank you so much for being willing to take part. According to a set of guidelines I have if a person I’m interviewing seems not fully aware of the interview or not able to take part in certain ways then I’m instructed to stop the interview. This is not your fault at all, and I really appreciate that you took time out for this schedule! I apologise it’s being cancelled! Put the interview on hold

- Take a note of the person’s ID
- Send an email to both email to Dr Stefan Rennick-Egglestone, and Professor Mike Slade informing them of the decision made to stop the interview.

If the person is deemed to have capacity carry on with interview.
